# Quantum secure direct communication: whispering with photons

**DOI:** 10.1093/nsr/nwaf096

**Published:** 2025-03-19

**Authors:** Min Wang, Gui-Lu Long

**Affiliations:** Beijing Academy of Quantum Information Sciences, China; Beijing Academy of Quantum Information Sciences, China; State Key Laboratory of Low-dimensional Quantum Physics and Department of Physics, Tsinghua University, China; Frontier Science Center for Quantum Information, China; Beijing National Research Center for Information Science and Technology, China

## Abstract

A comprehensive review of the latest advancements in quantum secure direct communication, and envision the establishment of an integrated space-ground quantum network in the future.

Quantum computing is advancing rapidly, posing a significant threat to information security, particularly to public-key cryptosystems like RSA. This has led to an urgent need for traditional cryptography to transition to post-quantum cryptography (PQC). In 2024, the National Institute of Standards and Technology (NIST) released the first batch of PQC standards and announced that quantum-vulnerable public-key schemes will be disallowed after 2035. Symmetric cryptography is less susceptible to known quantum attacks, and NIST-approved symmetric primitives with at least 128 bits of classical security are considered to meet the minimum security requirements for PQC standardization.

On the flip side, quantum mechanics offers a defense against quantum threats in the form of quantum communication. Its security is grounded in fundamental quantum principles such as superposition, entanglement and the no-cloning theorem. Quantum communication employs various technological means for different functions. For example, quantum teleportation enables the transfer of quantum states without physically moving the particles carrying those states between the sender and the receiver. Quantum key distribution (QKD) provides a solution for information-theoretically secure key establishment, while quantum secure direct communication (QSDC) ensures secure and reliable communication over noisy and eavesdropped channels by encoding information in quantum states.

The first QSDC protocol, the efficient protocol, was proposed in 2000. It allows direct communication using Einstein-Podolsky-Rosen (EPR) pairs without pre-establishing a key [1]. Information is encoded in EPR pairs and sent to the receiver in two batches, one qubit from each pair per batch. After the first batch is sent, security is checked for eavesdropping, and then the second batch is transmitted. Later, two single-qubit-based protocols were proposed [2,3]. In the DL04 two-way protocol [2], single photons are sent from the receiver to the sender first. Some qubits are randomly sampled for security checks, and then information is encoded using Pauli operations and sent back to the receiver. In the RECON one-way quasi-QSDC protocol [3], information is firstly encrypted with a pre-shared key, prepared on single-photon states and sent to the receiver. If no eavesdropping occurs, the shared key can be reused. Interestingly, Bennett *et al.* published a paper on quasi-QSDC in 2014, presenting the RECON protocol [4]. They designed the protocol as early as 1982, but its late publication was due to early rejections. They then focused on QKD due to its ease of implementation and robustness against high-loss quantum channels. The quasi-QSDC protocol preserves the transmission of information via quantum states, yet it still relies on a pre-shared key and classical one-time-pad encryption for security.

Experimental QSDC has been achieved using weak laser pulses with quantum characteristics [5], allowing legitimate parties to detect eavesdropping. When eavesdropping is detected, communication stops. Communication occurs only when no eavesdropping is detected, ensuring both the security of the information and the concealment of the communication action. If the communication is disrupted, legitimate users are alerted to stop and change their communication sites. As it is a form of communication, it is naturally compatible with the existing internet.

In recent years, QSDC has seen rapid theoretical and experimental progress. The SR3H theory, based on Wyner’s wiretap theory, has been established for secure and reliable communication in high-noise, high-loss and high-security-requirement channels. Using SR3H, channel loss and noise can be addressed while maintaining security. The one-hop QSDC distance has reached 100 km, making intercity QSDC feasible.

Networking is essential for practical applications. Long *et al.* [6] proposed secure relay networks, which are compatible with the conventional internet and feature end-to-end security with PQC, and outlined a seven-stage roadmap for a full-fledged quantum internet. In 2021, Qi *et al.* [7] demonstrated a 15-user QSDC network based on the efficient protocol of Long and Liu [1]. In 2022, Niu *et al.* [8] proposed a QSDC network scheme with optical switches to reduce terminal resources. In 2023, Wang *et al.* [9] experimentally demonstrated a functional three-node QSDC network with a secure relay. In 2024, Wang *et al.* [10] developed a quantum-channel access authentication scheme with comprehensive security features by encoding digital signatures in quantum states for transmission and verification. These advancements lay a solid foundation for large-scale quantum-secure communication networks.

Notably, while both QKD and QSDC are for security, they perform different tasks. QKD uses quantum principles to securely negotiate keys for symmetric cryptosystems, while QSDC encodes information directly onto quantum states for communication without pre-establishing a key. QKD extracts secure keys from noisy signals through privacy amplification (post-processing), while QSDC uses forward coding before transmission. QKD typically discloses all photon bases, while QSDC only discloses the bases of qubits used for eavesdropping checks. In access authentication and secure link establishment, QKD uses classical-communication-like authentication, while QSDC encodes signatures in quantum states for mutual authentication. In classical-repeater-linked networks, QKD requires trust relay nodes, while QSDC can use secure relays with classical encryption like PQC, and hence does not require the nodes to be trusted.

In conclusion, QSDC represents a new secure communication paradigm, transmitting information directly and securely using quantum states. In the near future, driven by advances in practical quantum state sources and detectors, as well as error-correcting codes and theoretical frameworks like the one-way and twin-field QSDC protocols, QSDC’s performance indicators such as distance, bandwidth and latency will improve, enabling the construction of practical metropolitan and intercity QSDC networks. QSDC will integrate with classical communication and cryptography to build secure and confidential networks, as shown in Fig. 1. In the long term, an integrated space-ground quantum communication network will connect quantum terminals, sensors and computers, forming a global quantum internet for worldwide secure communication, enhanced computing and advanced sensing.

**Figure 1.**
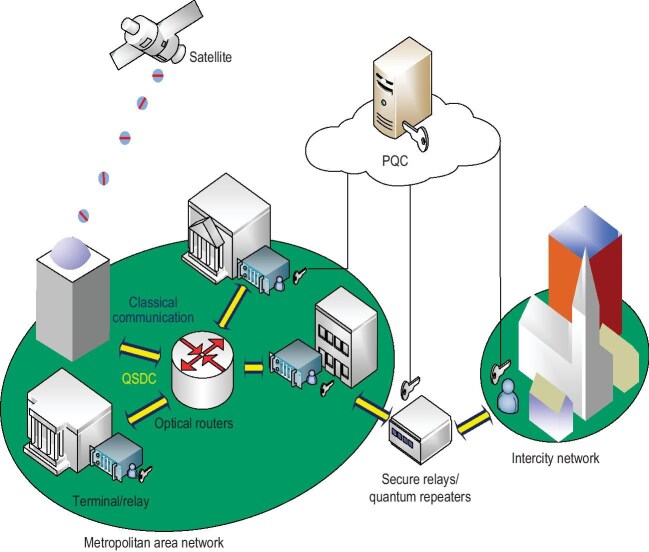
Diagram of a classic-quantum hybrid network.
